# Sublobar resection versus lobectomy in the treatment of synchronous multiple primary lung cancer

**DOI:** 10.1186/s12957-023-02996-w

**Published:** 2023-04-24

**Authors:** Niu Niu, Liang Zhou, Junjie Zhao, Xingjie Ma, Fan Yang, Weibo Qi

**Affiliations:** 1 Department of Cardiothoracic Surgery, First Hospital of Jiaxing (Affiliated Hospital of Jiaxing University), Jiaxing, 314000 China; 2 Graduate School of Bengbu Medical College, Bengbu, 233000 China

**Keywords:** Lobectomy, Sublobar resection, Synchronous multiple primary lung cancer

## Abstract

**Objective:**

Although synchronous multiple primary lung cancers (sMPLCs) are common in clinical practice, the choice of surgical modalities for the main lesion is still at the stage of exploration. This study is designed to analyze the prognosis of sMPLCs and single primary lung cancers with similar tumor stages and to explore whether sublobar resection has a similar prognosis as lobectomy for sMPLCs.

**Methods:**

One-hundred forty-one cases of sMPLCs were selected, including the following: 65 cases underwent lobectomy for main lesions, and 76 cases underwent sublobar resection for main lesions. One thousand one hundred forty-four cases of single primary lung cancer were matched at 1:1 by propensity score matching. Then, the patients with sMPLCs were divided into a lobectomy group and a sublobar group according to the first tumor stage. Ninety-eight cases of patients with sMPLCs were matched. The short-term perioperative effect, 5-year disease-free survival (DFS) rate, and 5-year overall survival (OS) rate between the two groups were compared.

**Results:**

There was no significant difference in OS between sMPLCs and single primary lung cancer after lobectomy (77.1% vs. 77.2%, *P* = 0.157) and sublobar resection (98.7% vs. 90.7%, *P* = 0.309). There was no significant difference in OS (86.7% vs. 83.9%, *P* = 0.482) or DFS (67.6 vs. 87.7%, *P* = 0.324) between the lobectomy group and sublobar group with sMPLCs. The sublobar resection group obtained a lower incidence of postoperative complications (40.8% vs. 16.3%, *P* = 0.007) and shorter postoperative hospital stay (11.22 vs. 9.27, *P* = 0.049).

**Conclusion:**

The prognosis of patients with sMPLCs generally depends on the main tumor state, which has no statistical difference regardless of sublobar resection or lobectomy, and the perioperative period of sublobar resection is safer than that of lobectomy.

## Key question

Is there a difference in the survival of sMPLCs and single primary lung cancers with similar tumor stages? Does sublobar resection have the similar prognosis as lobectomy for sMPLCs?

## Key findings

There was no difference in survival between sMPLCs and single primary lung cancer after lobectomy and sublobar resection. There was no significant difference in survival between the lobectomy group and sublobar group with sMPLCs.

## Take-home message

The prognosis of sMPLCs generally depends on its main tumor state. Whether sublobar resection or lobectomy, the prognosis of sMPLCs has no statistical difference.

## Introduction

In 1924, Beyreuthe [1] first reported two cases of independent lung cancer at the same time and introduced the concept of multiple primary lung cancer (MPLC) for the first time, that is, two or more primary lung malignant tumors occur simultaneously or successively in the same patient. According to the diagnosis interval of 2 years, it can be divided into synchronous MPLC (sMPLCs) and metachronous MPLC (mMPLC). MPLC was considered as a rare disease in the past. However, in recent years, due to the increasingly obvious trend of population aging, its detection rate is gradually increasing, and its incidence is on the rise, accounting for 3–13% of the total number of lung cancer cases [2–5].

Due to the differences in the characteristics of lung cancer, and more complex characteristics after the combination of multiple tumors, the difficulty of prognosis research of MPLC is greatly increased. Recently, most sMPLCs are diagnosed as early lung cancer, which can be cured in principle and have a good prognosis, but the results of prognostic studies are quite different [6–9]. The 2017 NCCN guidelines [10] recommend that standard lobectomy should be performed in patients with MPLC with sufficient pulmonary function reserve, while sublobar resection including segmentectomy resection and wedge resection can be used as an alternative. At present, among single-center studies, due to few cases, short follow-up time, less grouping, and other reasons, there is no reliable study on the relationship between surgical methods and the prognosis of sMPLCs, and whether the prognosis of sMPLCs depends entirely on the single staging of the first tumor is still lack of credible reports.

Therefore, this study conducted a single-center retrospective analysis to compare the efficacy of sublobar resection and lobectomy in the treatment of sMPLCs and to investigate whether the prognosis of sMPLCs is the same as that of matched single primary lung cancer, hoping to provide some references for the treatment of sMPLCs.

## Materials and methods

### Patients

This study retrospectively analyzed the clinical data of 2635 patients with non-small cell lung cancer (NSCLC) who underwent surgery in Jiaxing First Hospital from January 2012 to June 2019 and was approved by the Ethics Committee of Jiaxing First Hospital. Patients with sMPLCs with at least 2 lesions were screened out.

#### Entry conditions

In accordance with the diagnostic criteria of Martini and Melamed [11], sMPLC is as follows:

- (1) different pathological types; (2) multiple lesions found at the same time but located in different lobes and without lymph node or systemic metastasis; and (3) the same histological type but located in different segments, lobes, or bilateral lungs and originated from different carcinoma in situ; there was no cancer in the common lymph node drainage site and no extrapulmonary metastasis at the time of diagnosis.

The inclusion criteria of single primary lung cancer are as follows: (1) NSCLC with stages 1A–3A, (2) only one lesion, and (3) there was no cancer in the common lymph node drainage site and no extrapulmonary metastasis at the time of diagnosis.

**Fig. 1:**
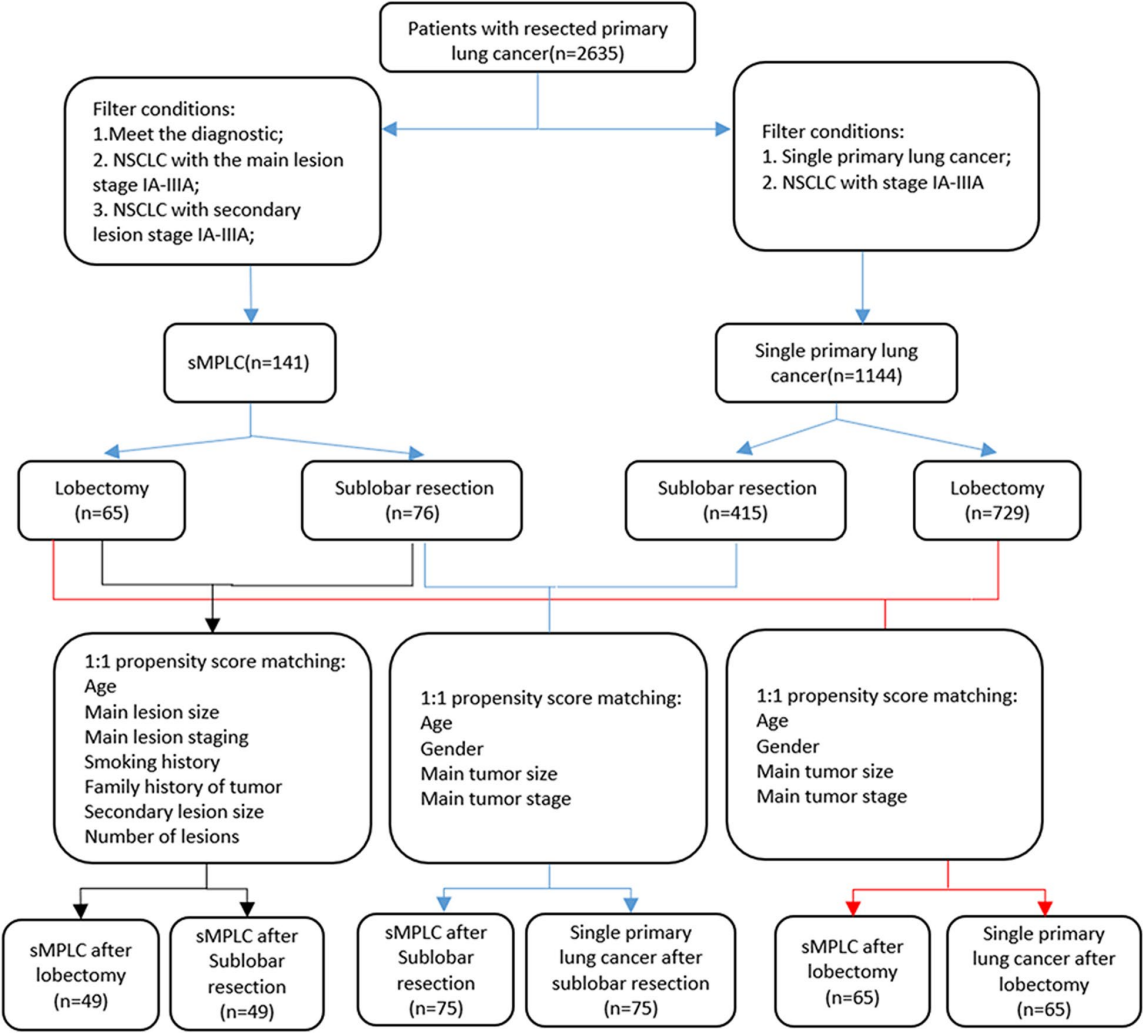
Screening and matching flowchart

A. According to the eighth edition of the international lung cancer staging standard, the main focus stage is the 1A–3A stage of NSCLC.
B. According to the eighth edition of the international lung cancer staging standard, NSCLC in which the secondary lesions were staged as stages 1A–3A, the loss of clinical data or survival follow-up time of less than 1 year was excluded. The detailed screening process is shown in Fig. 1.

Finally, 141 cases of sMPLCs with complete clinical data and postoperative survival follow-up data were selected, accounting for 5.3% of lung cancer patients in our hospital in the same period. The incidence rate is similar to the existing report [2–5]. In addition, a total of 1144 patients with single primary lung cancer were enrolled.

In order to balance the basic data of sMPLCs group and single primary lung cancer group, we performed the 1:1 tendency matching method according to the different surgical methods of the main tumor.

### Follow-up and related definition

All patients with sMPLCs had complete medical history and follow-up data. The main preoperative examinations were chest CT, abdominal color ultrasound, craniocerebral MRI or CT, and bone scan. The observation indicators included age, sex, preoperative complications, smoking history, and family history of lung cancer. The perioperative observation indexes included operation mode, operation time, postoperative drainage, postoperative hospital stay, postoperative complications, and hospitalization expenses. The postoperative pathological indexes included the size and stage of the main tumor, the size of the secondary tumor, the pathological type and the degree of pathological differentiation, and the time and location of recurrence or metastasis after operation. The follow-up time was from the operation day to the tumor recurrence, metastasis, death, or research cutoff date. Local recurrence was defined as lung recurrence and hilar lymph node and mediastinal lymph node recurrence on the operative side, and distant metastasis was defined as distant organ metastasis. The 5-year disease-free survival (DFS) was defined as the proportion of patients who had no tumor recurrence, metastasis, or cancer-related death from the end of the operation to the end of follow-up. The 5-year overall survival (OS) was defined as the proportion of patients who did not have cancer-related deaths from the end of the operation to the end of follow-up.

### Statistical methods

The data were processed by the *SPSS* 25.0 statistical software package, the measurement data were expressed by mean ± standard deviation (*x* ± *s*), and the propensity score matching was used to balance the basic data of the two groups of patients. *T*-test was used for data analysis among groups, percentage (%) was used for counting data, four-grid chi-square test was used for comparison between the groups, *Kaplan–Meier* survival curve was used for DFS analysis and OS analysis, and *log rank* was used for statistical analysis.

## Results

### Basic data statistics of patients with sMPLCs and single primary lung cancers

A total of 141 patients with sMPLCs who met the inclusion and exclusion criteria were divided into a lobectomy group (*n* = 65) and a sublobar resection group (*n* = 76). A total of 1144 patients with single primary lung cancer were enrolled in the group and divided into a lobectomy group (*n* = 729) and a sublobar resection group (*n* = 415). In order to balance the basic data of the two groups, we performed the 1:1 tendency matching method according to the different surgical methods of the main tumor, and the matching value was set at 0.01, which was 5% of the standard deviation of the tendency index. As a result, 65 pairs were matched in the lung lobectomy group, and 75 pairs were matched in the sublobar group. There were no significant differences in age, sex, and tumor stage between the two groups. The clinical features are shown in Tables 1 and 2.

**Table 1:** Pre-matching and after matching clinical characteristics of MPLC and single lung cancer main lesions in the lobectomy group

Factors	Pre-matching				After matching			
	sMPLC (*n* = 65)	SLC (*n* = 729)	*x*^*2*^*/t*	*P*	sMPLC (*n* = 65)	SLC (*n* = 65)	*x*^*2*^*/t*	*P*
Age, years ± SD	62.0±7.4	61.3±9.8	0.54	0.494	57.0±10.7	57.3±12.0	-0.22	0.892
Sex, n (%)								
Male	28 (43.1%)	371 (50.9%)	1.46	0.227	22 (28.9%)	137 (33.0%)	0.49	0.486
Female	37 (56.9%)	358 (49.1%)	0.99	0.912	54 (71.1%)	278 (67.0%)	2.263	0.150
Main tumor staging								
IA	51 (78.5%)	551 (75.6%)	0.67	0.516	72 (96.6%)	401 (97.3%)	0.262	0.793
IB	3 (4.6%)	46 (6.3%)			0 (0%)	2 (0%)		
IIA	3 (4.6%)	25 (3.4%)			1 (1.3%)	4 (0%)		
IIB	2 (3.1%)	35 (4.8%)			0 (0%)	1 (0%)		
IIIA	6 (9.2%)	72 (9.9%)			3 (4.0%)	7 (2.7%)		
Tumor size (mm)	22.0±12.5	23.3±14.8			12.1±7.6	11.8±9.3		

*SLC* single lung cancer

**Table 2:** Pre-matching and after matching clinical characteristics of MPLC and single lung cancer main lesions in the sublobar group

Factors	Pre-matching				After matching			
	sMPLC (*n*=76)	SLC (*n*=415)	*X*^*2*^*/t*	*P*	sMPLC (*n*=75)	SLC (*n*=75)	*x*^*2*^*/t*	*P*
Age, y ± SD	57.0±10.7	57.3±12.0	-0.22	0.892	62.0±7.4	60.8±9.9	0.79	0.432
Sex, n (%)								
Male	22 (28.9%)	137 (33.0%)	0.49	0.486	28 (43.1%)	23 (35.4%)	0.81	0.369
Female	54 (71.1%)	278 (67.0%)	2.26	0.150	37 (56.9%)	42 (64.6%)	3.33	0.504
Main tumor staging								
IA	72 (96.6%)	401 (97.3%)	0.262	0.793	51 (78.5%)	57 (87.7%)	0.67	0.507
IB	0 (0%)	2 (0%)			3 (4.6%)	1 (1.5%)		
IIA	1 (1.3%)	4 (0%)			3 (4.6%)	3 (4.6%)		
IIB	0 (0%)	1 (0%)			2 (3.1%)	2 (3.1%)		
IIIA	3 (4.0%)	7 (2.7%)			6 (9.2%)	2 (3.1%)		
Tumor size (mm)	12.1±7.6	11.8±9.3			22.0±12.5	20.4±15.3		

*SLC* single lung cancer

### Comparison of prognosis between sMPLC and single lung cancer

**Fig. 2:**
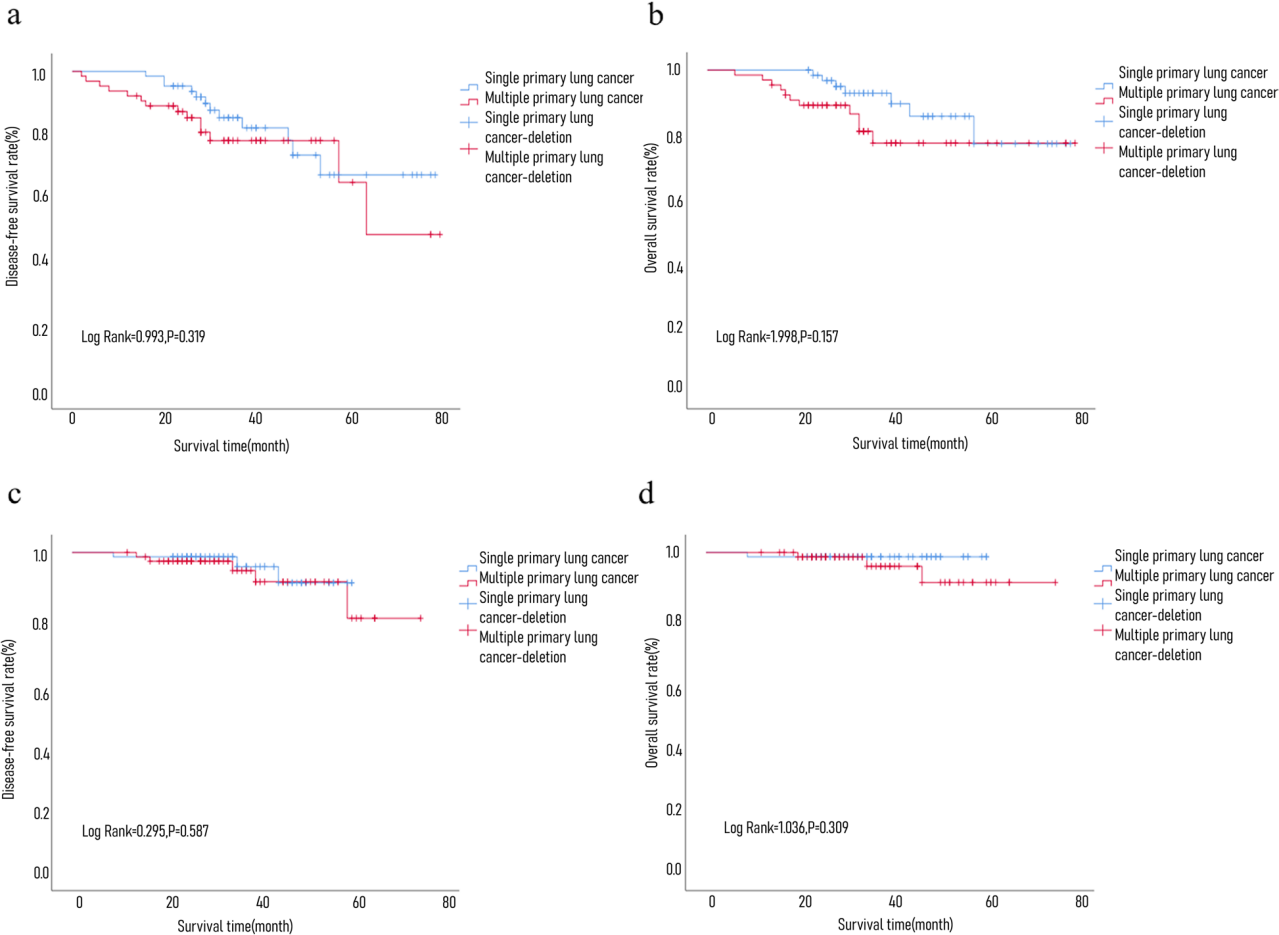
**a** Comparison of single primary lung cancer and sMPLC DFS after lobectomy. **b** Comparison of single primary lung cancer and sMPLC OS after lobectomy. **c** Comparison of single primary lung cancer and sMPLC DFS after sublobar resection. **d** Comparison of single primary lung cancer and sMPLC OS after sublobar resection

A total of 140 cases of single primary lung cancer were matched, including 65 pairs of lobectomy (including 65 cases in sMPLC group, 65 cases in single primary lung cancer group, and 664 cases in single primary lung cancer group were removed) and 75 pairs of sublobar resection (including 75 cases in sMPLC group, 75 cases in single primary lung cancer group; 1 case in sMPLC groups and 340 cases in single primary lung cancer group were removed). The patients in the two groups were followed up for 14–81 months. Up to the end of follow-up, 7 cases died after lobectomy for single primary lung cancer and 12 cases with recurrence and metastasis. Eleven cases died after lobectomy for sMPLCs and 14 cases with recurrence and metastasis. The 5-year DFS was 67.6 vs. 65.3%, *P* = 0.319. The 5-year OS was 77.1% vs. 77.2%, *P* = 0.157. A total of 1 case died of sublobar resection of single primary lung cancer; 3 cases of recurrence and metastasis; 3 cases of death of sublobar resection of multiple lung cancer; 5 cases of recurrence and metastasis; 5-year OS was 98.7% vs. 90.7%, *P* = 0.309; and 5-year DFS was 90.6% vs. 90.8%, *P* = 0.587. In the matched MPLC and single primary lung cancer, whether the main tumor underwent lobectomy or sublobar resection, the DFS and OS of the two groups were not significantly abnormal (Fig. 2).

### Basic data statistics of patients with MPLCs

#### General information of sMPLCs pre-matching and after matching

A total of 141 patients with sMPLCs were enrolled, including 50 males and 91 females. Their age ranged from 35 to 81, with an average of 55.8 years. The clinical characteristics of sMPLCs pre-matching and after matching with lobectomy and sublobar resection are shown in Table 3. In order to balance the basic data of the two groups of patients, we performed a 1:1 propensity matching method according to the different surgical methods of the main lesion (lobectomy and sublobar resection) in the sMPLCs group. The included parameters were age, sex, the primary lesion tumor size, primary lesion stage, number of lesions, secondary lesion size, smoking history, and family history of lung cancer. The matching value was set at 0.2, which was 20% of the standard deviation of the propensity index. The results matched 49 pairs of sMPLC lobectomy and sublobar resection, with a total of 98 cases (43 cases were excluded). Although there were still differences in the size of primary and secondary lesions after matching, the difference was decreasing, and there was no statistically significant difference in the stages of primary lesions and tumor differentiation between the two groups after matching. Due to the limitation of the number of cases, perfect propensity matching could not be achieved, but overall, there was no significant difference in age, sex, main lesion size and stage, degree of differentiation, number of lesions, and pathological type between the two groups of patients (*P* > 0.05).

**Table 3:** General information of sMPLCs before and after matching

Factors	Pre-matching				After matching			
	Lobectomy (*n* = 65)	Sublobar (*n* = 76)	*Χ*^2^*/t*	*P*	Lobectomy (*n* = 49)	Sublobar (*n* = 49)	*Χ*^2^*/t*	*P*
**Age, years ± SD**	62.0 ± 7.4	57.0 ± 10.7	3.25	0.001	61.6 ± 7.6	59.3 ± 10.6	1.18	0.243
**Sex, *n* (%)**			3.06	0.080			0.40	0.527
Male	28 (43.1%)	22 (28.9%)			19 (38.8%)	16 (32.7%)		
Female	37 (56.9%)	54 (71.1%)			30 (61.2%)	33 (67.3%)		
**Largest tumor size (mm)**	22.0 ± 12.5	12.1 ± 7.6	17.7	0.000	17.1 ± 7.4	13.57 ± 8.9	2.14	0.035
< 20 mm	29 (44.6%)	70 (92.1%)	38.8	0.000	29 (59.2%)	43 (87.8%)	10.26	0.001
≥ 20 mm	36 (55.4%)	6 (7.9%)			20 (40.8%)	6 (12.2%)		
**Secondary lesion size (mm)**	11.7 ± 6.4	7.7 ± 3.3	4.77	0.000	10.0 ± 5.6	8.3 ± 3.5	1.83	0.071
< 10 mm	26 (40.0%)	54 (71.1%)	15.2	0.000	25 (32.1%)	33 (66.7%)	2.12	0.146
≥ 10 mm	39 (60.0%)	22 (28.9%)			23 (59.2%)	16 (33.3%)		
**Tumor number**			0.99	0.608			1.26	0.532
2	54 (83.1%)	64 (84.2%)			40 (81.6%)	41 (83.7%)		
3	11 (16.9%)	11 (14.5%)			9 (18.4%)	7 (14.3%)		
≥ 4	0 (0%)	1 (1.3%)			0 (0%)	1 (2.0%)		
**Main lesion staging**			9.79	0.044			1.76	0.622
IA	51 (78.5%)	72 (94.8%)			43 (87.8%)	46 (93.9%)		
IB	3 (4.6%)	0 (0%)			0 (0%)	0 (0%)		
IIA	3 (4.6%)	1 (1.3%)			1 (2.0%)	0 (0%)		
IIB	2 (3.1%)	0 (0%)			1 (2.0%)	1 (2.0%)		
IIIA	6 (9.2%)	3 (3.9%)			4 (8.2%)	2 (4.1%)		
**Smoking**			1.2	0.273			0.08	0.779
Yes	13 (20.0%)	10 (13.2%)			7 (14.3%)	8 (16.3%)		
No	52 (80.0%)	66 (76.8%)			42 (87.5%)	41 (84.7%)		
**Family history of lung cancer**			0.01	0.918			0.71	0.399
Yes	4 (6.1%)	5 (6.6%)			2 (4.1%)	4 (8.2%)		
No	61 (93.9%)	71 (93.4%)			47 (95.9%)	45 (91.8%)		
**Histologic type**			6.43	0.169			7.12	0.870
Double adenocarcinoma	54 (83.1%)	71 (93.5%)			43 (87.8%)	45 (91.9%)		
Double squamous	8 (12.3%)	2 (2.6%)			4 (8.2%)	2 (4.1%)		
Adenocarcinoma-squamous	1 (1.5%)	1 (1.3%)			1 (2.0%)	1 (2.0%)		
Squamous-adenocarcinoma	1 (1.5%)	0 (0%)			0 (0%)	0 (0%)		
Other	1 (1.5%)	2 (2.6%)			1 (2.0%)	1 (2.0%)		
**Differentiation**			21.7	0.000			4.98	0.083
High	25 (38.5%)	57 (75.0%)			25 (51.0%)	34 (69.4%)		
Moderate	18 (27.7%)	13 (17.1%)			11 (22.4%)	10 (20.4%)		
Poorly	22 (33.8%)	6 (7.9%)			13 (26.6%)	5 (10.2%)		
**Complications**			3.69	0.297			7.49	0.112
0	35 (53.8%)	31 (40.8%)			25 (51.0%)	16 (32.7%)		
1	17 (26.2%)	31 (40.8%)			12 (24.5%)	23 (46.9%)		
2	11 (16.9%)	11 (14.5%)			9 (18.4%)	8 (16.3%)		
≥ 3	2 (3.1%)	3 (3.9%)			3 (6.1%)	2 (4.1%)		
**Simultaneous surgery**			5.25	0.022			7.51	
Yes	51 (78.5%)	46 (60.5%)			38 (77.6%)	25 (51.0%)		0.006
No	14 (21.5%)	30 (39.5%)			11 (22.4%)	24 (49.0%)		
**Second tumor surgery method**			37.7	0.000			15.68	
Lobectomy	36 (55.4%)	6 (7.9%)			24 (49.0%)	6 (12.2%)		
Segmentectomy	7 (10.8%)	17 (22.4%)			6 (12.2%)	12 (24.5%)		
Wedge	22 (33.8%)	53 (69.7%)			19 (38.8%)	31 (63.3%)		
**Chemotherapy**			5.01	0.025			1.10	0.294
Yes	11 (16.9%)	4 (5.2%)			6 (12.2%)	3 (6.1%)		
No	54 (83.1%)	72 (94.8%)			43 (87.8%)	46 (93.9%)		

#### The perioperative situation of two groups of sMPLCs

There were no deaths during the perioperative period of the enrolled sMPLCs. The operation time of lobectomy for sMPLCs, postoperative drainage, hospitalization expenses, and sublobar resection were not significantly different, but the hospital stay after lobectomy was longer than sublobar resection (Table 4).

**Table 4:** Comparison of perioperative indexes between lobectomy and sublobar resection

Factors	Lobectomy (*n* = 65)	Sublobar (*n* = 76)	*Χ*^2^*/t*	*P*
Operation time (min)	131.6 ± 58.8	123.9 ± 56.9	0.66	0.514
Postoperative hospital stay (day)	11.22 ± 5.35	9.27 ± 4.33	1.99	0.049
Drainage volume after 3 days (ml)	727.2 ± 328.9	654.5 ± 322.7	1.11	0.272
Respiratory tract infection			5.52	0.019
Yes	17 (34.7%)	7 (14.3%)		
No	32 (65.3%)	42 (85.7%)		
Respiratory failure			1.00	0.500
Yes	1 (2.0%)	0 (0%)		
No	48 (98.0%)	49 (100%)		
Pulmonary embolism			0.34	0.558
Yes	2 (4.0%)	1 (2.0%)		
No	47 (96.0%)	48 (98.0%)		
Total complications			7.20	0.007
Yes	20 (40.8%)	8 (16.3%)		
No	29 (52.0%)	41 (83.7%)		
Hospitalization expenses (¥)	45,892 ± 9311	43,789 ± 7856	1.21	0.230

#### Prognosis of sMPLCs undergoing lobectomy and sublobar resection

**Fig. 3:**
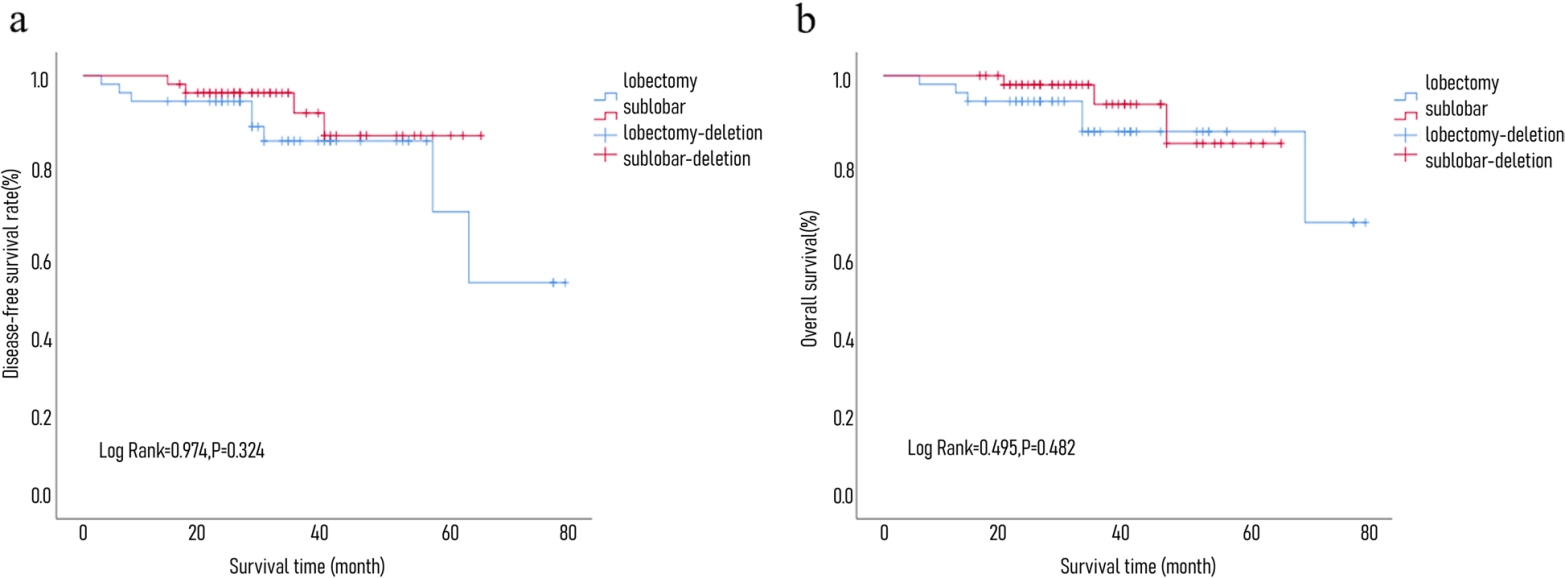
**a** Comparison of DFS in sMPLC groups: sublobar resection vs lobectomy. **b** Comparison of OS in sMPLC groups: sublobar resection vs lobectomy

A total of 49 pairs of patients were matched, with a total of 98 cases. Patients in the two groups were followed up for 12–81 months. By the end of the follow-up, 3 cases of the sublobar group patients died and 4 cases of recurrence and metastasis, 6 cases of patients died in lobectomy group and 8 cases of recurrence and metastasis, and all deaths and recurrence or metastasis were cancer related. DFS rate is as follows: the 1-year, 3-year, and 5-year DFS of the two groups were 93.9% vs. 98.0%, 84.4% vs. 91.1%, and 67.6 vs. 87.7%, *log rank* = 0.974, *P* = 0.324; the survival rates are as follows: 1-year, 3-year, and 5-year OS were 95.9% vs. 100%, 86.7% vs. 93.2%, and 86.7% vs. 83.9%, *log rank* = 0.495, *P* = 0.482. The DFS and OS of matched sMPLCs were not statistically significant regardless of sublobar resection or lobectomy (survival curve comparison is shown in Fig. 3.)

## Discussion

sMPLC was considered a rare disease in the past. In recent years, due to the continuous improvement of the diagnosis level, its incidence has increased. Existing research suggests that the incidence of MPLCs is on the rise. Guo [12] summarized 326 cases of MPLC with the incidence rate of 5.6%, and our result showed that the rate was 5.7%, which is similar to global research [5, 13–15] after the twentieth century. There are currently no clear diagnostic criteria for sMPLC, and the MM diagnostic criteria in 1975 [11] are the most classic. Of course, ACCP [16] guidelines have partially revised the MM criteria in 2003, 2007, and 2013, and the main point of the improvement is to increase the identification of differences in molecular genetics on the original basis. Dr. Liu Yi [17] found that the diagnostic criteria for MM and the second-generation gene sequencing technology based on gene rearrangement have a diagnostic consistency rate of 91.9% in the multicenter sMPLCs diagnostic verification test. Because gene sequencing technology has not been widely used clinically, the current MM standard is still the main reference standard for the diagnosis of MPLCs. Our research on the diagnosis of MPLCs also followed the MM standard in 1975. In this study, the pathological results of sMPLCs were mainly adenocarcinoma-adenocarcinoma, followed by squamous cell carcinoma-squamous cell carcinoma. However, different pathological types of sMPLCs were relatively rare, which is also in line with existing reports [12, 18, 19].

Many investigations have been carried out to help predict and improve the prognosis of lung cancer in recent years [20, 21]. For example, Le V. H. et al. [22] developed a model for predicting OS in patients with NSCLC based on risk scores of CT-based radiomics signatures. However, due to the complicated design of research involving sMPLC, there are still no multicenter prospective controlled studies to support and guide the treatment of this disease. At present, the treatment of sMPLCs only relies on the experience of clinicians and the consensus of a few experts. There are few studies on the prognosis regarding surgical treatment of MPLCs and single primary lung cancers. Early studies [23–29] have shown that lobectomy has a better prognosis than limited resection. In recent years, due to the overall diagnostic level of lung cancer improvement, the staging of operable lung cancer is relatively early, especially for the single ground-glass nodules with tumors ≤ 2 cm; sublobar resection can obtain a similar prognosis to lobectomy [30, 31]. Yu’s study [10] showed that there was no significant difference in the 5-year survival rate of MPLCs matching the single primary lung cancer stage (61.3% vs. 68.8%, *P* = 0.474). The sample size in our research was expanded. A total of 141 patients with sMPLC were enrolled in the group, and the grouping was more refined. We compared the prognosis of patients with sMPLCs and single primary lung cancers who underwent lobectomy or sublobectomy for main tumor, respectively. We used statistical methods to match single primary lung cancers with sMPLCs in terms of age, sex, and size of main tumor, especially similar in stage of main tumor, whether lobectomy was selected (77.1% vs. 77.2%, *P* = 0.157) or sublobar resection (98.7% vs. 90.7%, *P* = 0.309) can achieve similar oncological prognosis. This result also indicates that the prognosis of sMPLCs depends on its main tumor stage.

If the prognosis of multiple primary tumors depends on the staging of its main tumor, whether the surgical options for the main lesions of sMPLCs are the same as that of single primary lung cancer remains to be further studied. For single primary lung cancer, the North American Lung Cancer Research Group’s research [32] established the gold standard positioning of lobectomy + mediastinal lymph node dissection for the treatment of operable lung cancer more than 20 years ago. In recent years, due to changes in the types of lung cancer, more and more indolent lung cancers, sMPLCs, and elderly lung cancer patients who cannot tolerate lobectomy have gradually increased. Sublobar resection with less damage, including anatomical segmentectomy and wedge resection, is gradually increasing [33]. The current research [30, 34] supports anatomical segmentectomy for the treatment of stage 1 NSCLC, especially for stage 1 lung cancer with a diameter of ≤ 2 cm. These studies only focus on single primary lung cancer, and there is no prospective study on sublobar resection for sMPLCs. Previous retrospective studies [13–16] included few cases and did not specifically compare the prognosis of sMPLCs with different surgical methods. Trousse [13] believes that pneumonectomy alone is an independent risk factor for MPLC surgery. Yu [10] pointed out that for patients with stage 1 bilateral MPLC, sublobar resection can achieve a 5-year survival rate of 75%, not inferior to lobectomy. Xue [35] also believes that sMPLCs with two or more tumors should be evaluated separately and treated as independent tumors, and the prognosis of MPLCs is significantly better than that of metastatic lung tumors.

Because of the limitations of surgical access in the sublobar group, the diameter of the main lesion of sublobar group was smaller than that of lobectomy group, and the main lesion in the lobectomy group was staged relatively late. The sMPLCs are generally dominated by 2 lesions, and the pathological type is mainly adenocarcinoma-adenocarcinoma, which is also consistent with existing reports [12, 25, 30, 31, 10, 32, 33, 30, 34, 35]. Because carcinoma in situ often does not spread to local lymph nodes or distant metastases, the 5-year survival rate of patients with carcinoma in situ is close to 100%; while patients with invasive adenocarcinoma are more aggressive and prone to recurrence and metastasis after surgery, the prognosis is poor [36, 37]. In this study, we excluded patients whose main lesion was carcinoma in situ, and the 5-year OS rate of 141 patients with sMPLC was 84.6%, which was higher than some current retrospective studies [9, 10, 17, 30, 34], similar to Guo Haifa’s research results [12]. It is worth noting that this study suggests that different surgical methods for the main lesion do not affect the prognosis of sMPLCs. In order to further balance the bias between the MPLC group and the sublobar group, we performed a tendency-matching analysis of sublobar resection and lobectomy in the main tumor of sMPLCs. A total of 49 pairs of sMPLCs were matched. Although the size of the primary and secondary lesions after matching is still different, the difference is decreasing, and there was no statistically significant difference in the stage of the main lesions and the degree of tumor differentiation. Due to the limitation of the number of cases, perfect propensity matching could not be achieved. However, there were still no statistically significant differences in age, sex, main lesion size and stage, degree of differentiation, number of lesions, and pathological types between the two groups. On this basis, we compared the prognosis of patients with sMPLCs who underwent lobectomy and sublobar resection. The 1-year, 3-year, and 5-year DFS rates of the two groups were 93.9% vs. 98.0%, 84.4,% vs. 91.1%, and 67.6 vs. 87.7%, *P* = 0.324, and the 1-year, 3-year, and 5-year OS rates were 95.9% vs. 100%, 86.7% vs. 93.2%, and 86.7% vs. 83.9%, *P* = 0.482, indicating no statistically significant difference in the DFS rate and OS rate of the matched MPLC lesions regardless of sublobar resection or lobectomy. The possible reason is that the main lesion staging is caused by a larger proportion of stage 1 MPLC. As the number of cases increases, this result may change but for the main lesion stage 1 MPLC, and the choice of method is not a decisive factor affecting the prognosis.

At present, with the popularization of thoracic surgery techniques, the surgical method of sublobar resection, especially anatomical segment resection, is gradually becoming more mature [38]. At present, from a technical point of view, sublobar resection is safe and feasible to treat early invasive NSCLC [39]. In the current literature, 10% of these major complications reported in some prospective trials and large database analyses occur late. In a recently published randomized study [40] (CALGB/Alliance 140,503), the inpatient mortality after lobectomy for patients with suitable cardiopulmonary function was 1.1%, and the inpatient mortality after segmentectomy was 0.6%. This study found that the incidence rate of serious complications of sMPLCs such as pulmonary embolism (3/141, 2.1%), respiratory failure (1/141, 0.7%), but the more frequent occurrence is lung infection (24/141, 17.0%). Postoperative complications in the sublobar resection group (7 cases of respiratory infection, 0 case of respiratory failure, 1 case of pulmonary embolism) were significantly less than that of the lobectomy group (17 cases of respiratory infection, 1 case of respiratory failure, 2 cases of pulmonary embolism). There was no death during the perioperative period. In general, sublobar resection for sMPLCs has shorter hospital stays than lobectomy (9.72 ± 4.33 vs. 11.22 ± 5.35, *P* = 0.049) and a lower incidence of postoperative complications (16.3% vs. 40.8%, *P* = 0.007). We believe that the advantages of sublobar resection in the perioperative period are mainly because the wound area is smaller than that of lobectomy, and the exudation is less; at the same time, its perioperative complication rates were lower than that of lobectomy. Therefore, in patients with MPLCs, sublobar resection can be considered as an effective alternative to lobectomy in perioperative.

There is no statistically significant difference in the prognosis of sMPLCs, whether lobectomy or sublobar resection is selected for the main lesion, compared with the corresponding single primary lung cancer. The prognosis of sMPLCs generally depends only on the main lesion. This special form of lung cancer can be considered in the traditional TNM staging system according to the staging of the main lesion to predict the patient’s prognosis more accurately. The main focus of MPLC surgery can be selected according to the patient’s lung function. Sublobar resection or lobectomy, and sublobal treatment of sMPLC, is safer than lobectomy in the perioperative period and shorter postoperative hospital stay.

This study is a retrospective study of a single institution with a small sample size and a certain degree of bias. Due to the limitation of the sublobar resection entry criteria, the tumor diameter of the sublobar resection group is smaller than that of the lobectomy, older age, and more comorbidities, which are often incalculable confounding factors. Based on these factors, we tried our best to balance the confounding factors of tumor staging and tumor size by propensity matching analysis. We did not observe a difference in survival between the two groups. In addition, compared with lobectomy, sublobar resection has perioperative advantages such as shorter hospital stay and lower postoperative complications. Finally, it should be pointed out that the choice of surgical methods for MPLCs is still in the exploratory stage, and prospective studies are needed to further verify these observations.

## Data Availability

The data used to support the findings of this study are included within the article.
